# Design and Implementation of a Program Development Practicum for Faculty Education and Advancement of Clinical Programs

**DOI:** 10.3390/pediatric14040054

**Published:** 2022-10-31

**Authors:** Pam Ward, Henry C. Lin

**Affiliations:** Division of Pediatric Gastroenterology, Doernbecher Children’s Hospital, Portland, OR 97239, USA

**Keywords:** professional development, program management, project proposal

## Abstract

Physicians are often tasked to develop and lead collaborative, program development efforts but many have limited formal training. We designed and evaluated a professional development workshop series to provide our faculty members with a framework and tools for the development of clinical programs: the Program Development Practicum (PDP). Faculty identified a clinical program of focus and for each clinical program identified, a program proposal, SBAR communication (situation, background, assessment, recommendation), executive summary, 1-min elevator pitch, and budget was developed. Five clinical programs were identified for improvement including: Inflammatory Bowel Disease, Celiac Disease, Transition of Care, Integrative Health Clinic, and Endoscopic Procedures. At the conclusion of the PDP, these programs were presented to key hospital leaders and resulted in an investment of resource support. Faculty also reported increased understanding of overall program development with the largest gains in knowledge in proposal writing and marketing. Overall, the PDP allowed for a revamp of key clinical services and faculty clarity on resource availability and expectations. We plan to continue with annual engagement of hospital leaders to share updates.

## 1. Introduction

Similar to many academic subspecialty divisions, a strategic priority of our Division is to optimize clinical care for our patients through the continued development of “Centers of Excellence” for patients with complex medical conditions. These clinical centers embody a comprehensive care approach to not only provide high quality medical care and partner with local providers to create a medical home for these patients, but also provide access to relevant educational materials, clinical trials, and opportunities for community and patient engagement. Within our Division of Pediatric Gastroenterology at Doernbecher Children’s Hospital, previously identified medical conditions that would benefit from a Center of Excellence approach included inflammatory bowel disease, celiac disease, eosinophilic esophagitis, liver disease, motility disorders, short bowel disorders, and feeding and swallowing disorders. Our faculty members with expertise in these medical conditions have worked to establish infrastructure for these Centers of Excellence based on their prior experiences, but many have received minimal formal education in organizational dynamics and the nuances of program development, proposal writing, and program management. 

As healthcare becomes more complex, requiring interdisciplinary collaboration and communication, there is an expectation that physicians possess these requisite skills to be effective leaders.The challenge is that physicians are often tasked to develop and lead these collaborative efforts despite limited training. Existing leadership and professional development courses tend to focus more on general leadership approaches with limited practical application or formal evaluation of impact [1,2,3]. We realized that our academic physicians will be successful if they have the tools, resources and support needed to further develop these existing programs and “Centers of Clinical Excellence”. Being mindful of burnout and faculty morale, it was important to provide our faculty with dedicated time for such training without adding to their current clinical work.

**Figure 1:**
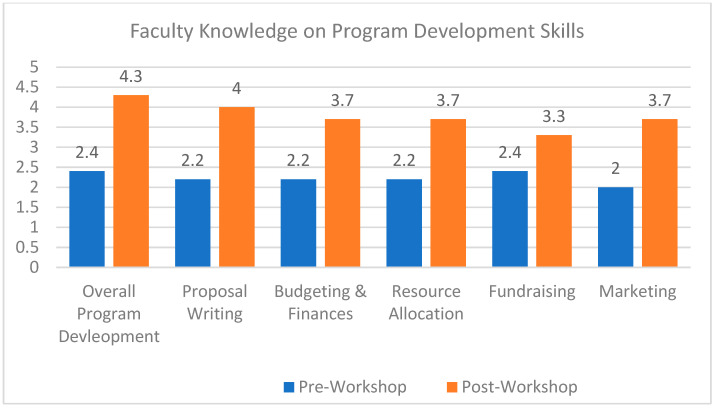
Faculty knowledge on program development skills. Faculty responses based on 5-point Likert scale with 5 being the most knowledge.

We designed and evaluated a professional development workshop series to provide our pediatric gastroenterology faculty members with a framework and tools for the development of clinical programs. The workshop content and overall curriculum was designed in collaboration with our partners across the Department of Pediatrics, including: ambulatory operations leadership, finance, strategic planning, philanthropy, provider relations, and marketing. Through engagement of these partners, common program design and implementation pitfalls, and physician knowledge gaps were identified. In addition, an initial knowledge gap analysis of the faculty was performed with faculty reporting some understanding of the various aspects of program development. The average faculty score was 2.4 out of 5, with 5 being very knowledgeable with program development (Figure 1). Based on these results and informed by adult learning theory, an interactive, 6-series workshop titled the Program Development Practicum (PDP), was developed to guide faculty through the following process: (1) problem identification, (2) proposal development, (3) facilitating interaction with stakeholders, and (4) proposal presentation. 

## 2. Materials and Methods

The Program Development Practicum (PDP) was developed to guide faculty through clinical program development and did not require faculty to have any prerequisite knowledge. However, faculty engagement in the PDP was integral as the PDP curriculum was designed around a “capstone” project in which faculty would have the opportunity for practical application of the skills and knowledge obtained from each workshop. At the start of the PDP, faculty were surveyed about their existing knowledge about program development. Each faculty also identified a specific clinical program that they were passionate about improving. The PDP challenged faculty to explore and update the clinical program based on their ideal best practices, with the goal of applying the PDP curriculum towards developing their updated clinical program.

**Figure A1:**
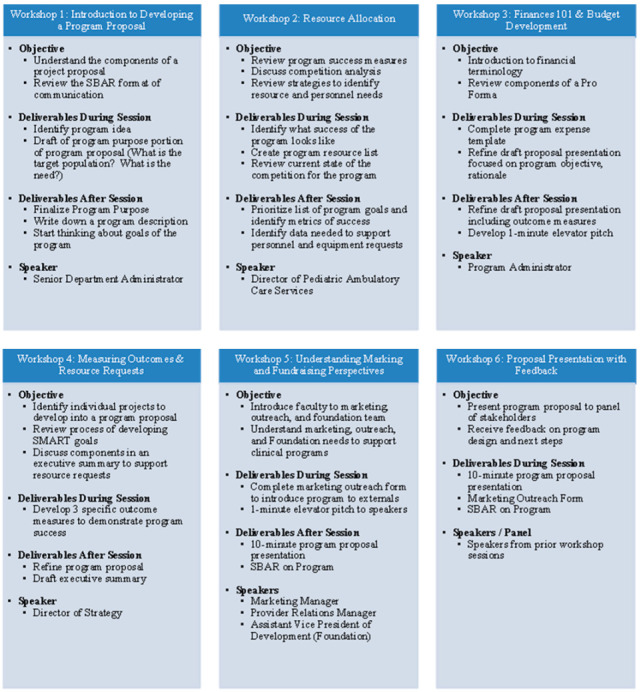
Program Development Practicum workshop sessions and deliverables.

Each 4-h workshop of the PDP had a theme that consisted of a presentation by a stakeholder content expert followed by explanation of key deliverables. Faculty were given time to work on these deliverables individually and in small groups during the each of the workshops, while also having the opportunity for individual coaching from the content expert. Workshop themes included: introduction to proposal writing, budgeting & finances, personnel & resource allocation, fundraising, and marketing initiatives to externals. The objectives and deliverables for each of the workshops were designed to build upon content developed in the previous workshop sessions (Figure A1). 

The first 3 workshops were designed to facilitate formulation of the idea, with a focus on what the faculty member would consider to be a success. Program administrators from our Department of Pediatrics Program Manager and the Director of Strategic Planning shared common challenges and pitfalls when introducing a clinical program and provided our faculty with an institutional framework for program development. Based on their vision of success, the faculty identified key stakeholders and perceived resource needs. The faculty were challenged to think about methods for achieving their goals with current available resources. As the resource list was refined, the faculty were led through a series of discussion to translate their vision of program success into what is important to their previously identified key stakeholders. Through this exercise of adopting different perspectives to think about their program success, faculty developed SMART (specific, measurable, achievable, relevant, and time-bound) metrics for success. 

At the start of the 4th workshop, faculty presented an overview of their updated clinical program, followed by a group discussion to determine which programs were ready for development into an official proposal. Five programs were identified including: Inflammatory Bowel Disease, Celiac Disease, Transition of Care, Integrative Health Clinic, and Endoscopic Procedures. The Inflammatory Bowel Disease and Endoscopic Procedures programs were updates of existing initiatives, while Celiac Disease, Transition of Care, and Integrative Health Clinic were new program ideas. Teams for each identified program were formed for the remainder of the PDP with the goal of developing the following content at by the conclusion of the PDP: A 10-min program proposal, SBAR communication (situation, background, assessment, recommendation), executive summary, 1-min elevator pitch, and budget.

The PDP culminated with a program proposal presentation during the 6th workshop to the key stakeholders within the Department of Pediatrics, many of whom were guest speakers at prior workshop sessions. The stakeholders provided real-time feedback on each program proposal. Evaluations were conducted for each workshop session and included an assessment on both the content as well as the speaker. Each workshop evaluation was reviewed by the PDP development team prior to the next workshop and adjustments to content were made based on current faculty progress and feedback received. There was also a final evaluation for the entire PDP. 

## 3. Results

The PDP was run from March to May 2021 with 2 workshops a month. Faculty received dedicated time to attend the PDP without the interruption of clinical duties. All faculty from our Division, which includes 9 physicians and 1 clinical psychologist, participated with experience ranging from junior faculty in practice for less than a year to faculty with over 20 years of clinical practice experience in an academic setting. At the completion of the PDP, faculty reported increased understanding of overall program development with an average score of 4.3 (Figure 1). The largest gains in knowledge were in faculty knowledge on proposal writing and marketing, but faculty reported gains across all domains of developing a program proposal.

In addition to improvement of faculty knowledge, the PDP facilitated the identification of the 5 aforementioned clinical programs for further development.: As part of the discovery process, faculty learned about different clinical practices, identified key expertise and perspectives to incorporate into the structure of the clinical program, and developed consensus about the priorities for each program. Faculty leaders for each program formally presented their program proposal and vision to key departmental leaders. These proposals followed the institution preferred format that was developed during the PDP, along with institution required supporting materials such as a SBAR, budget and marketing form. A Division-wide resource request was submitted based on the proposals with a successful outcome of both personnel and resource commitment to support these clinical programs. Specifically, the Division received personnel to help with administrative support and care coordination for the clinical programs, with some programs such as Endoscopic Procedures also receiving equipment to support programmatic growth.

Overall, faculty reported that they gained meaningful skills and developed hospital relationships. In particular, the PDP provided faculty a forum for skill development, dedicated time to collaborate with colleagues, and an opportunity to engage with hospital leaders. Interactions with hospital stakeholders in the learning environment offered by the PDP allowed faculty to develop a shared understanding of each stakeholder’s responsibilities and how to partner more effectively, strengthening professional relationships. Faculty shared their clinical passion and learned to articulate how programmatic investments would not only benefit the patient, but also help advance key institution initiatives. Perhaps just as notable is that despite some interruptions in clinical service to allow faculty to have dedicated time for the PDP, there was no negative impact on clinical productivity or to patient access

## 4. Discussion

The initial concept of the PDP was to provide faculty with the knowledge base, tools, and time to develop a clinical program or professional passion. Anecdotally, physicians are often tasked with developing clinical programs with limited formal training. In the absence of dedicated guidance, despite well-intentioned attempts to develop or improve upon an existing idea, it can be difficult to successfully navigate hospital dynamics. Institutions, like ours, may offer mentorship or leadership courses, but these may be limited in scope or dedicated time for participation. Others still may seek out specific training to bridge their knowledge gap such as by pursuing leadership courses or in some cases, a business administration degree. Ultimately, there is limited published literature on program development or project management training implementation and evaluation for physicians. 

Part of the motivation for designing and investing in the PDP course was in response to improving physician engagement and morale. Prior work by our Division had identified an ongoing cultural desire to feel valued and to embrace professional satisfaction while providing high quality care. The dueling focus on quality care versus clinical productivity can create a dynamic in which physicians may feel misalignment with the institutional operations or objectives. There may also be a perceived lack of input on clinical strategy, which can lead to confusion or frustration if a clinically meaningful investment in patient care resources is unable to be supported at a given point in time. Strategies to help with physician engagement may help to reduce these negative feelings as well as burnout. As such, hospital leaders have a responsibility for helping their team members better understand and navigate these organizational dynamics [4,5].

The negative consequences of physician burnout or disengagement on patient safety and overall quality of care including patient satisfaction have been well documented. Burnout can also affect personal health. This amalgam of factors may contribute to physician turnover, resulting a replacement cost to the patient as well as for the institution. Physician engagement in the work environment is one strategy to help address burnout [6,7,8]. The PDP was created in response to assessing and acknowledging faculty desire for dedicated training. The PDP was developed as a specific intervention based on this feedback. Acknowledging time constraints in the busy academic and clinical schedule, we integrated the program into the faculty schedule by cancelling clinical assignments if they interfered with a workshop time. We rotated workshop days as an attempt at equity among cancelling clinics. This time and resource investment not only helped us show appreciation for our faculty, but also allowed us to algin our Division’s values and continue to cultivate the sense of community [8].

The overall result from the PDP was a revamp of key clinical services and faculty clarity on resource availability and expectations. A year after the PDP, following the successful resource request, each of the identified clinical programs has continued to develop under the stewardship of the faculty leader. An example of success has been an accessible multidisciplinary pediatric inflammatory bowel disease clinic that supports over 350 children. Despite this initial success, continual program assessment is necessary. As such, we have planned for an annual engagement with hospital leaders to share updates and any potential investments needed for our clinical programs using the tools learned from the PDP.

Limitations of this study include the small sample size to allow for adequate assessment of the impact of the PDP curriculum. While the speakers and content of the PDP should be reasonably straightforward to replicate, there is a degree of buy-in necessary to remove clinical expectations to facilitate participation in the PDP. In review of PDP participant feedback, having dedicated time was key to engagement as this investment signaled the importance of participation and helped faculty feel valued. However, with institutional support and faculty engagement, the curriculum of the PDP can be applied for other academic institutions for faculty professional development and we have shared the PDP model with other groups at our institution.

As our clinical programs continue to evolve, we launched a follow-up Project Management Practical Course for our faculty to learn the strategies and tools for developing and advancing a task plan. The goal of this follow-up course is to engage faculty in conversations to explore barriers and potential solutions to bringing plans to fruition. Based on feedback from the PDP, we have identified the following learning outcomes: (1) give and receive support for delivering on the clinical program leadership task, (2) identify components of a task plan, articulate them, and begin to execute on the task, and (3) grow an understanding of individual learning styles as well as the promise cycle and practice using it to advance a task. We also aim to host a mini-PDP workshop every few years to allow faculty dedicated time to revisit their clinical programmatic passions and assess for ideas that are ready for further development. 

## Data Availability

Data is contained within the article.
